# The Surgical Section of the Rochester Academy of Medicine

**Published:** 1912-03

**Authors:** 


### The Surgical Section of the Rochester Academy of Medicine

Met February 14, 1912 at the Genesee Valley Club. Dr. Thomas S. Cullen of Baltimore discussed Diseases of the Umbilicus, other than Hernia; his brother Dr. Ernest Cullen of Detroit, spoke on the Test of Renal Function in Surgical Disease of the Kidney.

